# Childhood physical abuse and subsequent violent victimization among people who use illegal drugs in Vancouver, Canada

**DOI:** 10.1371/journal.pone.0255875

**Published:** 2021-08-12

**Authors:** Julie Sagram, William Lee, JinCheol Choi, M.-J. Milloy, Kanna Hayashi, Kora DeBeck, Evan Wood, Thomas Kerr

**Affiliations:** Division of Social Medicine, Department of Medicine, British Columbia Centre on Substance Use, University of British Columbia, Vancouver, British Columbia, Canada; Nagoya University Asian Satellite Campuses Institute, Nagoya University Graduate School of Medicine, JAPAN

## Abstract

**Background:**

Violent victimization is common among people who use illegal drugs (PWUD) and is a source of significant health-related harm. However, little attention has been paid to how antecedent childhood trauma among PWUD may contribute to the risk of victimization in adulthood.

**Objective:**

This study sought to examine the relationship between childhood physical abuse and victimization by physical assault among adult PWUD.

**Participants and setting:**

Data were derived from three prospective cohorts of PWUD in Vancouver, Canada between 2005 and 2018.

**Methods:**

Childhood physical abuse was assessed using the Childhood Trauma Questionnaire. Generalized linear mixed modeling was used to estimate the relationship between childhood physical abuse and subsequent violent victimization, after adjustment for potential confounders.

**Results:**

Among 2960 PWUD, including 1018 (34.39%) female participants, 1030 (34.8%) participants reported a history of moderate to severe childhood physical abuse, and 949 (32.06%) participants reported recent violent victimization at baseline. In a multivariate analysis, childhood physical abuse (Adjusted Odds Ratio [AOR] = 1.34, 95% confidence interval [CI]: 1.23–1.46) remained independently associated with violent victimization after adjustment for potential confounders.

**Conclusions:**

This study found a high prevalence of childhood physical abuse and that this was associated with a higher risk of subsequent violent victimization among PWUD in this setting. Greater support for PWUD with a history of childhood physical abuse is needed to decrease existing vulnerability to violence, including screening for and treatment of childhood trauma and related violence prevention.

## Introduction

Violence among people who use illegal drugs (PWUD) is common and is associated with elevated risks for significant health and social harm. Many past studies, from a range of international settings, have shown that drug use can increase the risk of both violent perpetration and victimization [1, 2]. The pervasiveness of violence among PWUD has been explained in part by Goldstein’s framework [3] that describes the role of psychopharmacological, economic, and structural factors in perpetuating violence among PWUD. This model suggests that violence may be influenced by drug-induced states such as hyperactivity or reduced vigilance (i.e., pharmacological factors), committing violent crime such as robbery to obtain drugs or money for drugs (i.e., economic factors), and the inherent dangers associated with the illegal drug trade (i.e., systemic factors) [3]. Solutions to reduce and prevent violence among PWUD have come mostly in the form of increased law enforcement [4]. However, recent evidence suggests that enforcement-based interventions have potential to exacerbate violence in the population [5, 6]. Given the harmful outcomes associated with violence at both the individual and community-level, there is a need to identify and intervene upon other factors that serve to perpetuate violent victimization, including those that go beyond the pharmacological and structural factors outlined above.

One factor that may play a role in perpetuating violence is childhood trauma, although this has not received much attention in the literature on violence among PWUD. Given that exposure to childhood maltreatment increases the risk of developing a substance use disorder (SUD) later in life [7], it is not surprising that childhood trauma is common among adult PWUD [8, 9]. Indeed, the connection between childhood trauma and SUD has been well-documented [10–12], and has been explained using the self-medication theory [13], which suggests that individuals may develop a SUD by treating or muting negative psychological symptomology with drugs or alcohol [13]. For example, the numbing or euphoric effects of opioids can be effective in temporarily relieving the psychological distress experienced from posttraumatic stress disorder (PTSD) [14]. This theory has been supported by studies that show the effect of trauma cues on drug cravings [15, 16] and the effectiveness of trauma-focused therapy in decreasing substance use [16, 17].

While there has been a growing number of studies done in the last two decades examining the role that childhood trauma plays in adult victimization [18–21], little attention has been given to the specific role that childhood physical abuse may play in perpetuating violent victimization among adult PWUD. There is also a need to examine the phenomenon of revictimization as it relates to non-sexual physical assault to help fill the gap in revictimization literature (which has historically focused on sexual revictimization) by studying the experiences of individuals who report histories of non-sexual childhood physical abuse, as revictimization typically occurs within the same category of violence [22]. Accordingly, this study sought to investigate whether childhood trauma as a result of physical abuse is associated with recent violent victimization among a cohort of PWUD in Vancouver, Canada.

## Methods

### Participants and procedures

This study draws upon longitudinal data collected between December 2005 and November 2018 from three ongoing, open prospective cohort studies of PWUD in Vancouver, Canada: the Vancouver Injection Drug User Study (VIDUS), the AIDS Care Cohort to evaluate Exposure to Survival Services (ACCESS), and the At-Risk Youth Study (ARYS). VIDUS is a study of HIV-negative adult PWUD who have reported any injection drug use in the previous month before study enrolment. ACCESS is a cohort of HIV-positive adult PWUD who have reported any illegal drug use (other than or in addition to cannabis, which was legalized in October, 2018) in the month before enrolment. Finally, ARYS is a cohort study of street-involved youth, aged 14–26 years at the time of recruitment, who have reported any illegal drug use (other than or in addition to cannabis) in the past month. At baseline and semi-annually, study participants answer an interviewer-administered questionnaire that elicits information about socio-demographic characteristics, drug-use patterns, engagement with health-care services and law enforcement, and other behavioural and contextual factors. The three studies employ harmonized data collection to allow for combined analyses. Participants are recruited through extensive outreach methods, provide written and informed consent, and are compensated $40 for each study visit. Participants are asked to provide biological samples, including urine testing for initial drug screens, blood samples for HIV and HCV serological testing, or HIV clinical monitoring as appropriate. The studies have received approvals from the University of British Columbia Providence Health Care Research Ethics Board (UBC PHC REB).

### Measures

The outcome of interest was violent victimization, which is measured at baseline and semi-annually, and assessed by asking participants to respond yes or no to the following question: “In the last six months, have you been physically attacked or suffered any kind of physical violence, including torture or punishment related to a drug debt?”. In this study, violent victimization does not include sexual assault, which is treated as a distinct exposure, and the sample is restricted to those over the age of 18, since we are examining re-victimization in adulthood.

Our primary explanatory variable was childhood physical abuse, which is ascertained from the Childhood Trauma Questionnaire (CTQ), a reliable self-report measure with a Cronbach alpha of 0.9 in the current study. Participants are asked to complete the CTQ at their baseline interview. This instrument is a clinical screening tool with 28 items used to assess three forms of childhood abuse (sexual, physical, and emotional) and two forms of childhood neglect (physical and emotional). The CTQ provides a score for each of the five subscales that correspond to each type of abuse and neglect, based on responses to five items. It uses a five-point Likert scale ranging from “never true” (1) to “very often true” (5). For this current study, our explanatory variable was measured using only the childhood physical abuse subscale. Within this category of childhood abuse, participants respond to statements such as “When I was growing up… I got hit or beaten so badly that it was noticed by someone like a teacher, neighbour, or doctor”, “I believe that I was physically abused”, and “I was punished with a belt, a board, a cord (or some other hard object)” [23]. All questions refer only to events that occurred during childhood. Each subscale produces scores ranging from 5 to 25. We used recommended and pre-determined cut-off scores to translate the data on physical abuse into one of four levels of childhood trauma: none or minimal (5–8), low to moderate (9–12), moderate to severe (13–15), and severe to extreme (>15) [24]. Consistent with previous studies [23, 25] for this analysis, we chose to collapse the four levels into two: none to low (5–12) and moderate to extreme (13–25).

Variables considered as potential confounders were: age, sex at birth (male vs. female), sexual orientation (heterosexual vs. non-heterosexual), ethnicity (white vs. Black, Indigenous, or Other People of Colour [BIPOC]), homelessness in the last six months (yes vs. no), currently being in a relationship (no vs. yes), sex work in the last six months (yes vs. no), drug dealing in the last six months (yes vs. no), drug or alcohol treatment of any kind in the last six months, including: detox/youth detox, daytox, recovery house, treatment centre, counsellor, NA/CA/AA/SMART, methadone/methadose, Suboxone treatment, slow-release oral morphine, injectable opioid agonist treatment, alcohol relapse prevention medications, Onsite, residential community, out-patient treatment, drug treatment court, and “other” (no vs. yes), daily stimulant use in the last six months (i.e. methamphetamine, cocaine, crack, or prescribed stimulant use) (yes vs. no), daily illegal opioid use in the last six months (yes vs. no), and current heavy alcohol use (defined by the National Institute on Alcohol Abuse and Alcoholism as > 14 drinks per week or > 4 drinks on one occasion for men, and > 7 drinks per week or > 3 drinks on one occasion for women) (yes vs. no). All time-varying variables are time-updated and refer to the six-month period prior to follow-up interview, unless otherwise stated. All behavioural variables refer to the six-month period prior to follow-up interview and are treated as time-varying variables, unless otherwise stated.

### Statistical analyses

In bivariate analyses, we examined the baseline relationships between moderate to extreme childhood physical abuse and our variables of interest using Pearson’s chi-square test for categorical variables and Mann-Whitney’s U test for the continuous variable of age. We then used a generalized linear mixed model (GLMM) with a logit-link function to determine whether recent violent victimization was independently associated with childhood physical abuse after adjustment for confounders. We included all variables associated with the outcome in bivariate analyses at p < 0.1 into a multivariate model and used a stepwise approach to fit a series of reduced models. After comparing the coefficient value associated with the main outcome of interest in the full model to the coefficient value in each of the reduced models, we dropped the secondary variable associated with the smallest relative change. We continued this iterative process until the minimum change exceeded 5%. Remaining variables were considered confounders in the multivariate analysis. All statistical analyses were performed using R version 3.5.2 (R Foundation for Statistical Computing, Vienna, Austria). All p-values are two sided.

## Results

Data from 2960 PWUD were included in this study. Among this sample, 1018 (34.39%) were female, 1393 (47.06%) were BIPOC, and the median age was 32.1 years (Interquartile Ratio [IQR]: 23.26–43.46). The number of participants who reported a history of moderate to extreme childhood physical abuse was 1030 (34.8%) and 949 (32.06%) participants reported recent violent victimization (being physically assaulted in the last six months) at baseline. Of the 949 participants who reported physical assault, 399 (42.04%) were also victims of childhood physical abuse. In 16,597 interviews between 2005 and 2018, recent violent victimization was reported in 3329 (20.1%) interviews. Characteristics of the study sample stratified by violent victimization at baseline are presented in Table 1.

**Table 1:** Baseline characteristics of people who use illegal drugs in Vancouver, Canada, stratified by violent victimization (N = 2960).

Characteristic	Violent victimization*		*p—*value
	Yes (32%)	No (68%)	
	n = 949	n = 2011	
**Childhood physical abuse**			
Moderate to severe	399 (42.04)	631 (31.38)	<0.001
None to low	550 (57.96)	1380 (68.62)	
**Cohort**			
ACCESS	148 (15.6)	524 (26.06)	<0.001
ARYS	476 (50.16)	626 (31.13)	
VIDUS	325 (34.25)	861 (42.81)	
**Age**			
Median (IQR)	25.83 (22.21–39.84)	34.52 (24.05–44.99)	<0.001
**Sex**			
Male	662 (69.76)	1279 (63.6)	0.001
Female	287 (30.24)	731 (36.35)	
**Sexual orientation**			
Heterosexual	776 (81.77)	1677 (83.39)	0.194
Non-heterosexual	171 (18.02)	321 (15.96)	
**Ethnicity**			
White	394 (41.52)	1005 (49.98)	<0.001
BIPOC^+^	554 (58.38)	999 (49.68)	
**Homelessness** *			
Yes	598 (63.01)	902 (44.85)	<0.001
No	347 (36.56)	1099 (54.65)	
**In a relationship**			
No	296 (31.19)	613 (30.48)	0.727
Yes	644 (67.86)	1379 (68.57)	
**Sex work** *			
Yes	168 (17.7)	319 (15.86)	0.224
No	778 (81.98)	1687 (83.89)	
**Drug dealing** *			
Yes	488 (51.42)	713 (35.45)	<0.001
No	459 (48.37)	1296 (64.45)	
**Received drug or alcohol treatment** *			
No	504 (53.11)	1069 (53.16)	1
Yes	444 (46.79)	941 (46.79)	
**Daily stimulant use** *			
Yes	544 (57.32)	1207 (60.02)	0.176
No	404 (42.57)	802 (39.88)	
**Daily opioid use** *			
Yes	372 (39.2)	821 (40.83)	0.423
No	577 (60.8)	1190 (59.17)	
**Heavy alcohol use**			
Yes	356 (37.51)	581 (28.89)	<0.001
No	590 (62.17)	1429 (71.06)	

*Activities in the previous 6 months

^+^ Black, Indigenous or other People of Colour

In bivariate analysis, childhood physical abuse was positively associated with subsequent violent victimization (Odds Ratio [OR] = 1.33, 95% confidence interval [CI]: 1.21–1.46, *p <* 0.001). Other factors associated with recent violent victimization in bivariate analyses included: belonging to the ARYS cohort (OR = 2.28, 95% CI: 2.03–2.57), belonging to the VIDUS cohort (OR = 1.27, 95% CI: 1.13–1.43), younger age (OR = 0.97, 95% CI: 0.96–0.97), male sex (OR = 1.25, 95% CI: 1.13–1.38), being heterosexual (OR = 1.20, 95% CI: 1.06–1.36), white ethnicity (OR = 1.32, 95% CI: 1.21–1.45), homelessness in the last six months (OR = 1.90, 95% CI: 1.76–2.04), sex work in the last six months (OR = 1.29, 95% CI: 1.15–1.44), drug dealing in the last six months (OR = 1.74, 95% CI: 1.61–1.87), receiving any kind of drug or alcohol treatment in the last six months (OR = 0.92, 95% CI: 0.85–0.99), and current heavy alcohol use (OR = 1.24, 95% CI: 1.14–1.35).

As shown in Table 2, in our multivariate analysis, two variables (being heterosexual and receiving any kind of drug or alcohol treatment in the last six months) did not retain significance. Childhood physical abuse remained independently associated with subsequent violent victimization, after adjustment for other potential confounders (Adjusted Odds Ratio [AOR] = 1.34, 95% CI: 1.23–1.46, *p <* 0.001).

**Table 2:** Generalized linear mixed-effects analysis of factors associated with violent victimization among people who use illegal drugs in Vancouver, Canada (N = 2960).

Characteristic	Unadjusted		Adjusted	
	Odds Ratio (95% CI)	*p*—value	Odds Ratio (95% CI)	*p—*value
**Childhood physical abuse**				
(Moderate to severe vs. None to low)	1.33 (1.21–1.46)	<0.001	1.34 (1.23–1.46)	<0.001
**Cohort**				
(ARYS vs. ACCESS)	2.28 (2.03–2.57)	<0.001	1.19 (1.01–1.39)	0.034
(VIDUS vs. ACCESS)	1.27 (1.13–1.43)	<0.001	1.18 (1.05–1.32)	0.004
**Age**				
(Per year older)	0.97 (0.96–0.97)	<0.001	0.98 (0.97–0.99)	<0.001
**Sex**				
(Male vs. Female)	1.25 (1.13–1.38)	<0.001	1.32 (1.20–1.46)	<0.001
**Sexual orientation**				
(Heterosexual vs. Non-heterosexual)	1.20 (1.06–1.36)	0.004	-	-
**Ethnicity**				
(White vs. BIPOC^+^)	1.32 (1.21–1.45)	<0.001	1.17 (1.07–1.27)	<0.001
**Homelessness** *				
(Yes vs. No)	1.90 (1.76–2.04)	<0.001	1.45 (1.34–1.57)	<0.001
**In a relationship**				
(No vs. Yes)	1.00 (0.92–1.08)	0.922	-	-
**Sex work** *				
(Yes vs. No)	1.29 (1.15–1.44)	<0.001	1.25 (1.11–1.40)	<0.001
**Drug dealing** *				
(Yes vs. No)	1.74 (1.61–1.87)	<0.001	1.52 (1.41–1.64)	<0.001
**Received drug or alcohol treatment** *				
(No vs. Yes)	0.92 (0.85–0.99)	0.033	-	-
**Daily stimulant use** *				
(Yes vs. No)	1.06 (0.98–1.15)	0.123	-	-
**Daily opioid use** *				
(Yes vs. No)	0.95 (0.87–1.03)	0.196	-	-
**Heavy alcohol use**				
(Yes vs. No)	1.24 (1.14–1.35)	<0.001	1.26 (1.16–1.37)	<0.001

*Activities in the previous 6 months

^+^ Black, Indigenous or other People of Colour

## Discussion

In the present study, we found that childhood physical abuse was common among PWUD, with approximately one third (34.8%) of the sample reporting moderate to extreme childhood physical abuse. Likewise, almost one third (32.06%) of PWUD reported violent victimization in the last six months at baseline. In a multivariate longitudinal analysis, we found that childhood physical abuse remained positively associated with recent violent victimization after adjustment for a range of potential confounders, with those who experienced moderate to extreme childhood physical abuse having approximately one and a half times higher odds of violent victimization.

To our knowledge, this is the first study to examine the relationship between recent violent victimization and childhood physical abuse among PWUD. While most research on violence and substance use attributes violence to the psychoactive effects of drugs, structural factors, or the consequences associated with the prohibition of illegal drugs [26–28], our findings highlight the possible developmental and psychological antecedents of violent victimization among this population.

One proposed explanation for why revictimization occurs in these cases is the psychological effect that childhood abuse in general has on early life, as described by object relations theory [29]. This model suggests that during childhood, repeated abuse perpetrated by a caregiver forms a collection of deleterious perceptions and expectations of relationships, which are subsequently activated in adulthood [30]. Due to its typically repetitive and prolonged nature [31], childhood abuse in all forms can promote the development of complex PTSD [32], which in turn, has been widely linked to a heightened risk of revictimization [33]. Related symptoms include self-doubt, difficulty perceiving and responding to threats, expectation of pain and injury in relational contexts, and dissociation [29, 34, 35]. These psychological adaptations to the difficult circumstances of childhood physical abuse can unfortunately lead to self-destructive behaviour in adulthood that further increases the risk of violent victimization.

An alternative and equally compelling explanation for why childhood physical abuse may lead to future revictimization is the neurological damage precipitated by trauma [36]. Due to the neuroplasticity of the brain in early life [37], studies have found all forms of childhood abuse to be linked to the underdevelopment of the left hemisphere and hippocampus, which negatively affects logical thinking and memory [38]. Additionally, childhood abuse is linked to: diminished right-left hemisphere integration, which results in slower processing of information and cognitive avoidance [38, 39]; abnormalities in the cerebellar vermis, which plays an important role in regulating attention and emotion [38]; and dysfunction of the temporolimbic system [38], which impairs one’s judgement of others’ emotional states [40]. These negative brain adaptations could promote the risk of being victimized via a decreased ability for decision-making related to self-protection.

This study has limitations. First, the cohorts included in this study are community-recruited and not random samples, therefore the findings may not represent trends in the general population of people who use drugs. Second, our data was collected via self-reports and therefore response biases may have affected participant responses. Specifically, in regard to childhood trauma, reports may be influenced by a recall bias wherein individuals who repeatedly suffer difficult events throughout their lives are more likely to remember their adverse childhood experiences [41]. While some may be unable to recall memories of early victimization, others (for example, those reporting recent physical assault) may have stronger memories of childhood abuse, due to the perceived relevance of these events and a tendency to re-tell these stories in a search for meaning [41]. Finally, this study was observational in nature and therefore causality cannot be inferred from its findings.

Collectively, these findings support the need for a preventative, victim-centered, and trauma-informed approach to reducing the high rate of violent victimization among PWUD. Screening this population for childhood trauma in healthcare and other relevant facilities is recommended. Interventions such as trauma-focused cognitive behavioural therapy (TF-CBT) should be provided, as it has been proven to be an effective treatment for reducing the complex PTSD symptoms suffered by victims of childhood abuse [42]. It is important that such counselling services be made easily accessible for PWUD, given its potential to lower the rate of violence among PWUD by treating its associated psychological factors.

In conclusion, this study found that about one-third of surveyed PWUD in Vancouver, Canada have a history of childhood physical abuse, and that this form of trauma was independently and positively associated with subsequent violent victimization in adulthood. Future research should seek to elucidate the specific psychological mechanisms through which childhood trauma increases the likelihood of victimization in adulthood. Additionally, PWUD should be given greater access to evidence-based interventions (such as TF-CBT) that are aimed at improving safety for individuals with histories of childhood abuse.
